# Circulating microRNA profiles in plasma: identification of miR-224 as a novel diagnostic biomarker in hepatocellular carcinoma independent of hepatic function

**DOI:** 10.18632/oncotarget.10781

**Published:** 2016-07-22

**Authors:** Wataru Okajima, Shuhei Komatsu, Daisuke Ichikawa, Mahito Miyamae, Tsutomu Kawaguchi, Shoji Hirajima, Takuma Ohashi, Taisuke Imamura, Jun Kiuchi, Tomohiro Arita, Hirotaka Konishi, Atsushi Shiozaki, Ryo Moriumura, Hisashi Ikoma, Kazuma Okamoto, Hiroki Taniguchi, Yoshito Itoh, Eigo Otsuji

**Affiliations:** ^1^ Division of Digestive Surgery, Department of Surgery, Kyoto Prefectural University of Medicine, Kawaramachihirokoji, Kamigyo-ku, Kyoto, 602-8566, Japan; ^2^ Department of Surgery, Kyoto Second Red Cross Hospital, Haruobicho, Kamigyo-ku, 602-8026, Kyoto, Japan; ^3^ Department of Molecular Gastroenterology and Hepatology, Kyoto Prefectural University of Medicine, Kawaramachihirokoji, Kamigyo-ku, Kyoto, 602-8566, Japan

**Keywords:** plasma, circulating microRNA, liquid biopsy, liver cirrhosis, tumor marker

## Abstract

**Aims:**

This study was designed to identify novel microRNAs (miRNAs) in plasma for detecting and monitoring hepatocellular carcinoma (HCC), independent of hepatic function and background liver diseases with different etiologies.

**Results:**

(1) Four oncogenic miRNAs (miR-151, 155, 191 and 224) with high expression in HCC tissues were selected as candidates. (2) Quantitative RT-PCR using plasma samples from 107 HCC patients and 75 healthy volunteers revealed a significantly higher level of plasma miR-224 in HCC patients than in healthy volunteers according to a small-scale analysis (P < 0.0001), two independent large-scale cohort analysis (P < 0.0001, AUC 0.908). (3) miR-224 expression was significantly higher in HCC tissues and HCC cell lines than in normal hepatic tissues and fibroblasts, respectively. (P = 0.0011, 0.0150) (4) Plasma miR-224 reflected tumor dynamics; preoperative plasma levels of miR-224 were significantly reduced in postoperative samples (P = 0.0058), and plasma miR-224 levels were significantly correlated with paired miR-224 levels in HCC tissues (P = 0.0005). (5) Furthermore, plasma miR-224 levels significantly discriminated HCC patients from patients with chronic liver disease (P = 0.0008). A high plasma miR-224 level was significantly correlated with larger tumor size (P = 0.0005) and recurrences (P = 0.0027). The plasma miR-224 level could accurately detect small tumors less than 18 mm preoperatively.

**Methods:**

We performed a systematic review of the NCBI database and selected candidate miRNAs reported as highly expressed in HCC tissue.

**Conclusions:**

Plasma miR-224 may be a sensitive biomarker for screening HCC and monitoring tumor dynamics.

## INTRODUCTION

Hepatocellular carcinoma (HCC) is the second most common cause of cancer deaths worldwide. Despite continuous global efforts aimed at eradication and improvements in various treatment techniques, the prognosis of HCC remains poor. Even in major advanced economies, the mortality rates have been increasing [1]. Although HCC is a typical viral infection-related malignancy derived from chronic hepatitis B and C [2–3], HCC has also been strongly associated with lifestyle. Excessive alcohol consumption, obesity and type 2 diabetes are strongly associated with the carcinogenesis and development of HCC [4–7]. Thus, not only the proportion but also the number of HCC patients with non-viral etiologies has been increasing on a global scale [2].

Because identifying clinical biomarkers and molecular targets for HCC may contribute to improving the survival rate of patients with this lethal disease, several recent studies have clarified that certain molecules and associated signaling pathways, such as Ras/Raf/MAPK, PI3K/Akt/mTOR, Wnt-β-catenin, Hedgehog, HGF/c-Met and EGFR, have important roles in hepatocarcinogenesis [8–13]. In clinical settings, however, few molecules have been validated as diagnostic, therapeutic and/or prognostic biomarkers for HCC. Alpha-fetoprotein (AFP), AFP lectin fraction (AFP-L3) and proteins induced through vitamin K deficiency or antagonist-II (PIVKA-II), which is also known as des-γ-carboxy prothrombin (DCP), have been used as conventional serum tumor markers. These markers, however, lack sufficient sensitivity and specificity [14–16]. Hence, the use of less invasive technology for the development of novel molecular biomarkers for HCC, independent of background liver function and diseases with different etiologies, is needed and might facilitate the detection of early stage HCC and monitoring of tumor dynamics.

MicroRNAs (miRNAs), small non-coding RNAs that regulate the translation of specific protein-coding genes, were discovered in 1993 [17]. Since then, miRNAs have been intensively studied in cancer science. The altered expression of miRNAs has been associated with several diseases and implicated in the development of various cancers [18–21]. Recently, several studies have detected miRNAs in the plasma/serum in a remarkably stable form [19, 22–25]. Tumor-derived miRNAs are resistant to endogenous ribonuclease activity in plasma/serum, as these molecules bind to certain proteins, such as the Argonaute 2 protein and high-density lipoproteins [26–27], or are packaged into secretory vesicles, including apoptotic bodies and exosomes in plasma/serum [22, 28–30]. The expression of each miRNA in the blood is consistent in all healthy individuals [22–23]. Furthermore, secretory vesicles, containing specific miRNAs, function as intercellular transmitters; secreted miRNAs from donor cells can be transferred to and function in recipient cells [31–33]. These findings have prompted the identification of novel liquid biomarkers of miRNAs in cancers. We have previously identified cancer-associated miRNAs in plasma, which might be useful for the detection of cancer, monitoring of tumors, prediction of malignant potential, and prognosis and chemoresistance in gastric, esophageal and pancreatic cancers [25, 34–44].

Concerning plasma/serum miRNAs in HCC, several research groups have reported the potential utility of miRNAs circulating in plasma/serum in clinical applications [45–47]. However, these miRNAs are not necessarily candidates for HCC, and more sensitive and promising candidates for the early detection and treatment of HCC, independent of background liver diseases and liver function, are needed in clinical settings. Therefore, the aim of the present study was to identify novel plasma miRNA candidates using a genome-wide database approach.

In this study, we conducted a systematic review of the NCBI database and compared the plasma levels of four candidate miRNAs (miR-151, 155, 191 and 224) with an oncogenic function, reported as highly expressed in HCC tissue, between HCC patients and healthy volunteers. We ultimately validated plasma miR-224 as a useful biomarker for screening HCC, monitoring tumor dynamics and evaluating tumor residue following non-surgical treatment. Our results provide evidence that the plasma level of miR-224 meaningfully contributes to clinical decision-making.

## RESULTS

### Study design to find novel plasma miRNA biomarkers for HCC

**Figure 1:**
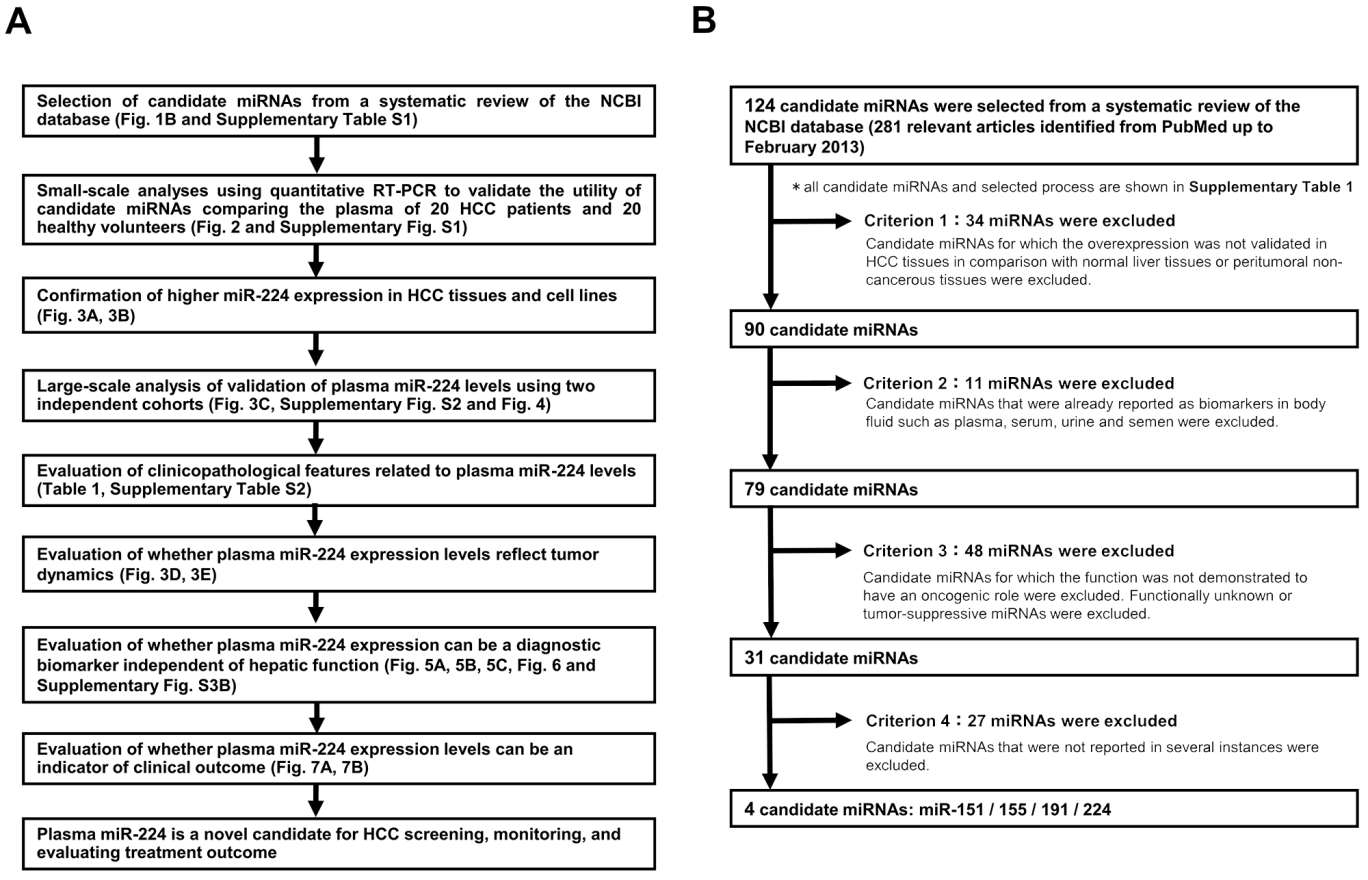
**A.** Selection of the candidate microRNAs from the NCBI database. **B.** Study design to identify novel plasma miRNA biomarkers for HCC. Using a systematic review of the NCBI database, we selected four candidate miRNAs for cancer detection and monitoring. Candidate miRNAs, previously reported to have an oncogenic role in HCC and previously confirmed in tissues or cell lines but not in body fluids, were selected. Among these, the expression level of plasma miR-224 showed the most significant difference (P < 0.0001). Thus, further analyses of plasma miR-224 were conducted.

**Figure 2:**
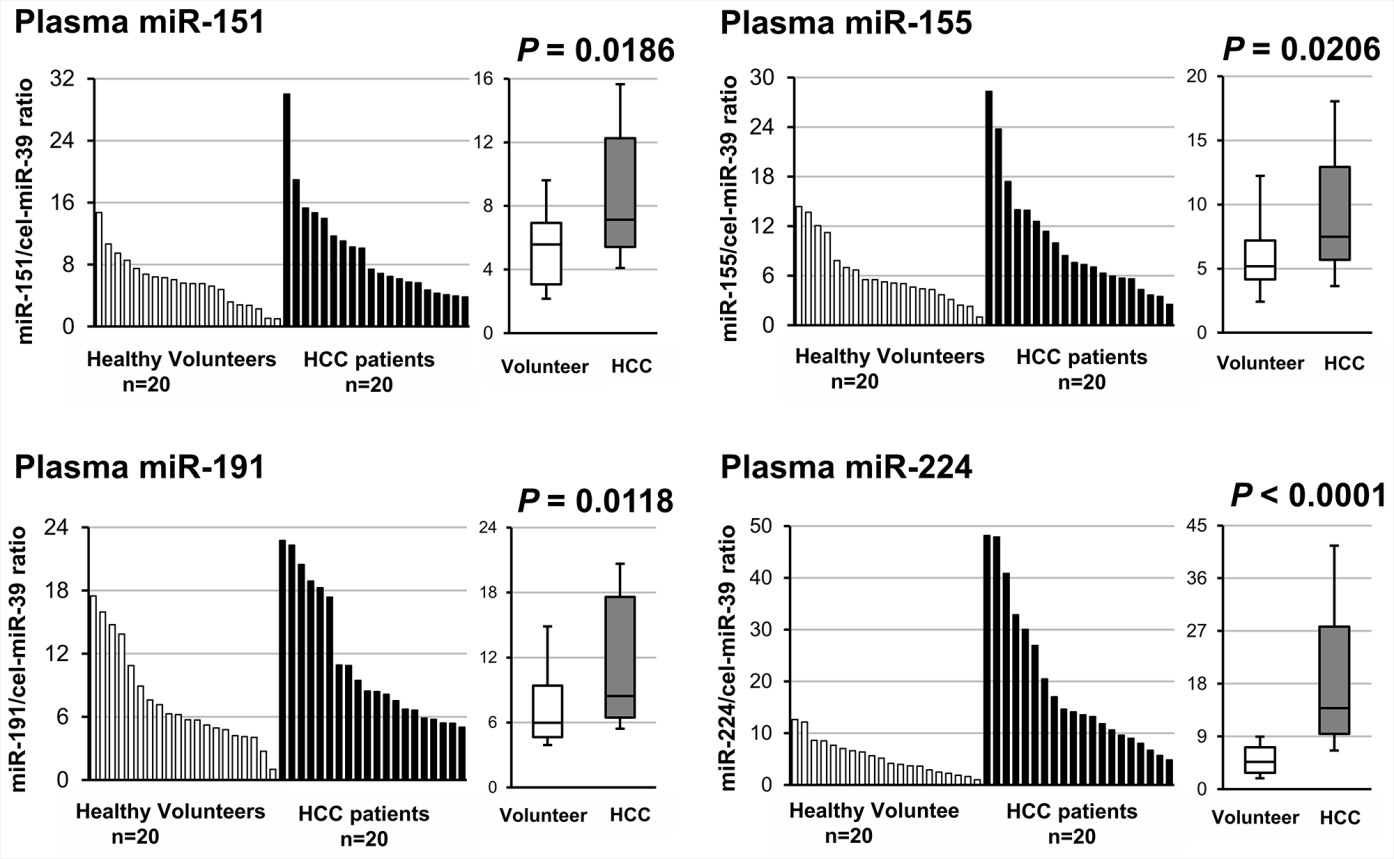
Small-scale analyses comparing the plasma levels of four candidate miRNAs between HCC patients and healthy volunteers Plasma levels of the four candidate miRNAs in 20 HCC patients and 20 healthy volunteers were analyzed using qRT-PCR. The expression level of each miRNA was normalized to that of cel-miR-39 as described in the Materials and Methods. All candidate miRNAs showed significantly higher expression levels in HCC patients than in healthy volunteers. Among these, the expression level of plasma miR-224 showed the most significant difference (P < 0.0001).

**Figure 3:**
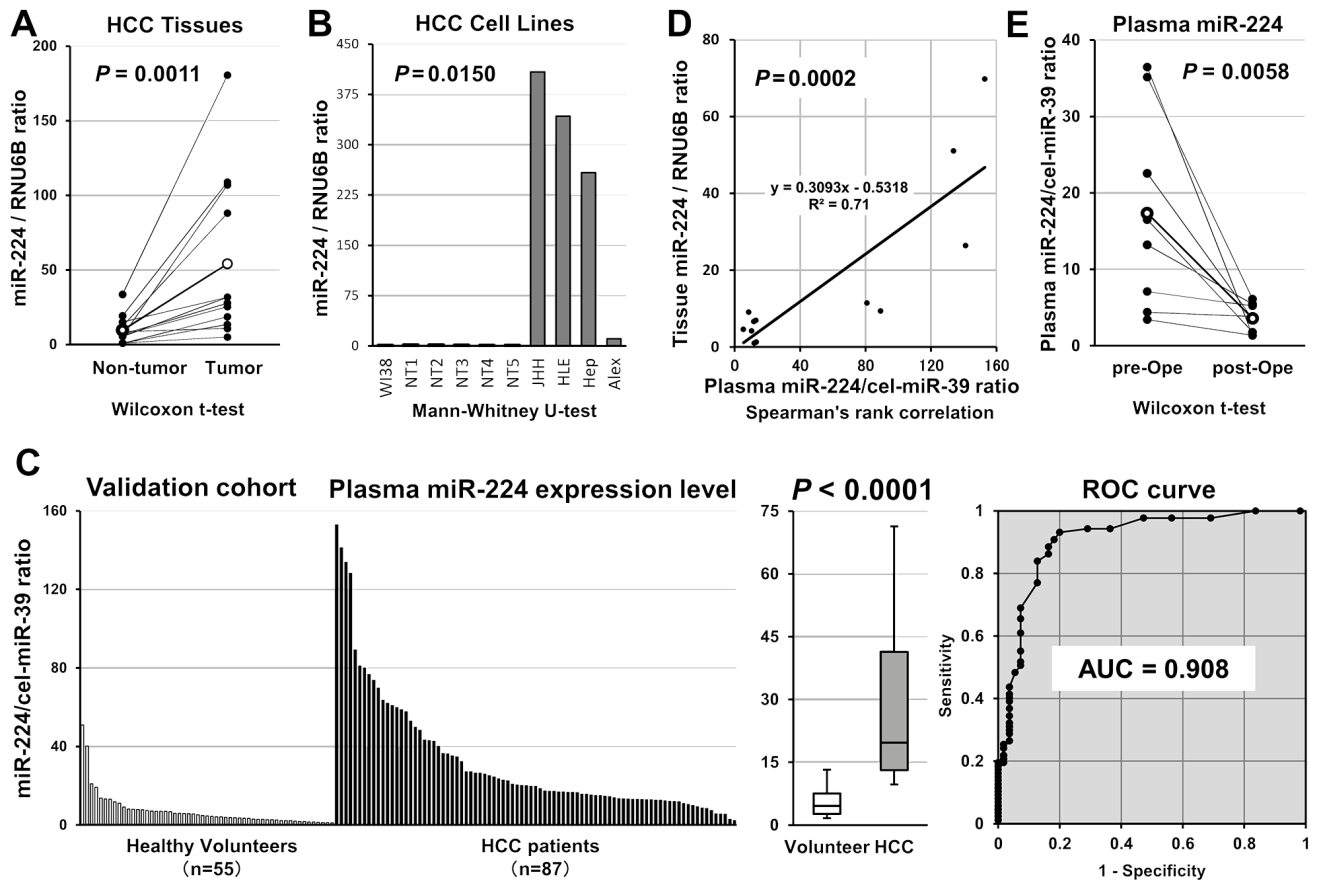
**A, B.** Confirmation of the expression level of miR-224 in HCC tissues and HCC cell lines. miR-224 expression was significantly higher in HCC tissues (P = 0.0011) and HCC cell lines (P = 0.0150) than in normal tissues and fibroblasts, respectively. **C.** Large-scale analysis of the plasma level of miR-224 in HCC patients using a validation cohort. The expression level of plasma miR-224 was significantly higher in HCC patients than in healthy volunteers (P < 0.0001), and this finding was validated in large-scale analysis (AUC was 0.908). For the large-scale analysis, total RNA extracted from plasma samples obtained from 87 HCC patients and 55 age-matched healthy volunteers were used to analyze the expression level of miR-224 using qRT-PCR. **D.** The correlation between the plasma and the tissue levels of miR-224 were confirmed in HCC patients using paired samples (P = 0.0005). **E.** Evaluation of whether the plasma miR-224 levels reflect tumor dynamics. The expression level of miR-224 was significantly reduced in postoperative plasma samples (P = 0.0058).

**Figure 4:**
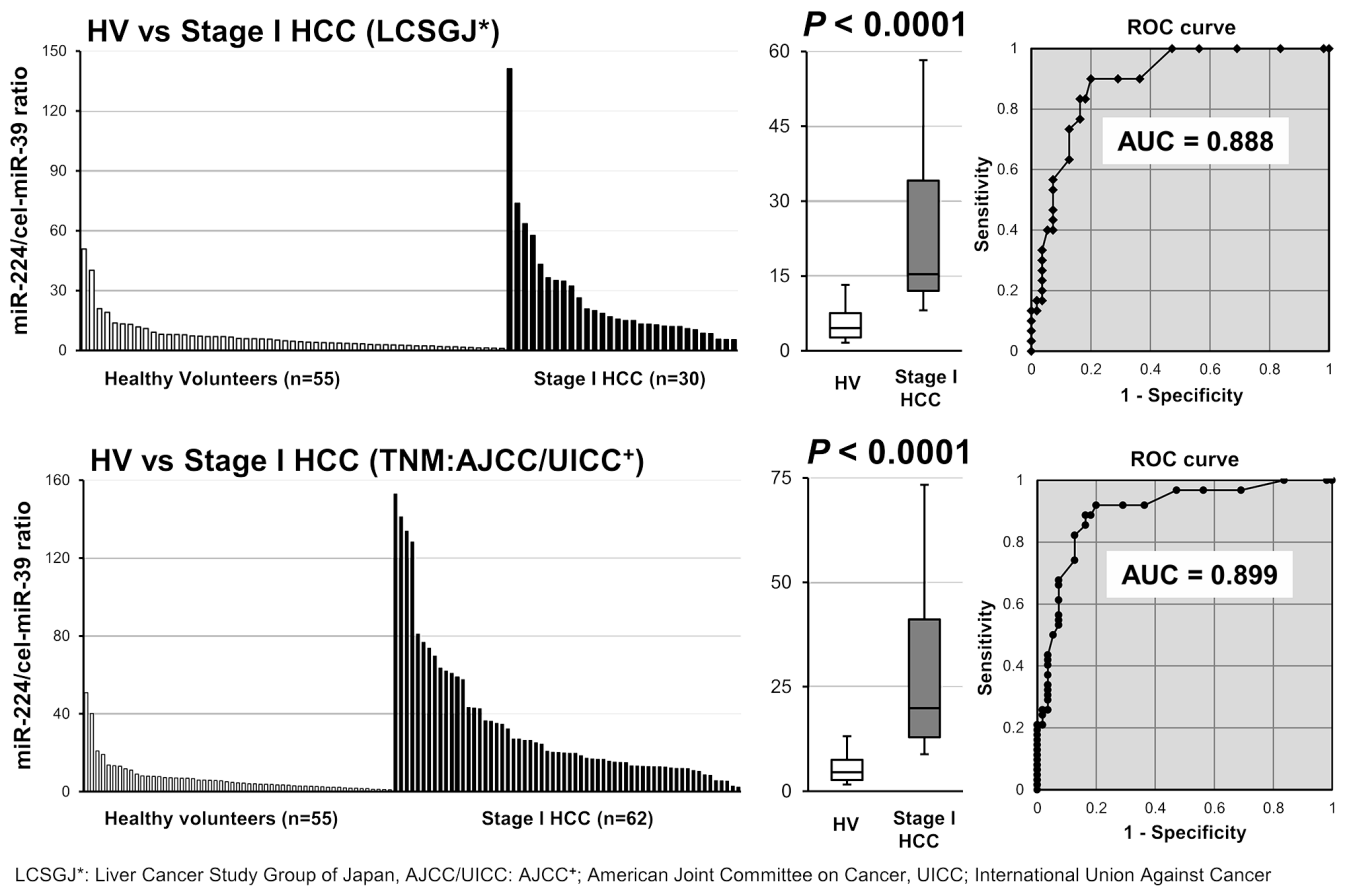
Evaluation of whether the plasma miR-224 level could satisfactory distinguish early stage HCC patients form healthy volunteers The expression level of plasma miR-224 was significantly higher in stage I HCC patients than in healthy volunteers (P < 0.0001). The AUC values in the LCSGJ and TNM (AJCC/UICC) staging systems for the plasma miR-224 analysis were 0.888 and 0.899, respectively.

**Figure 5:**
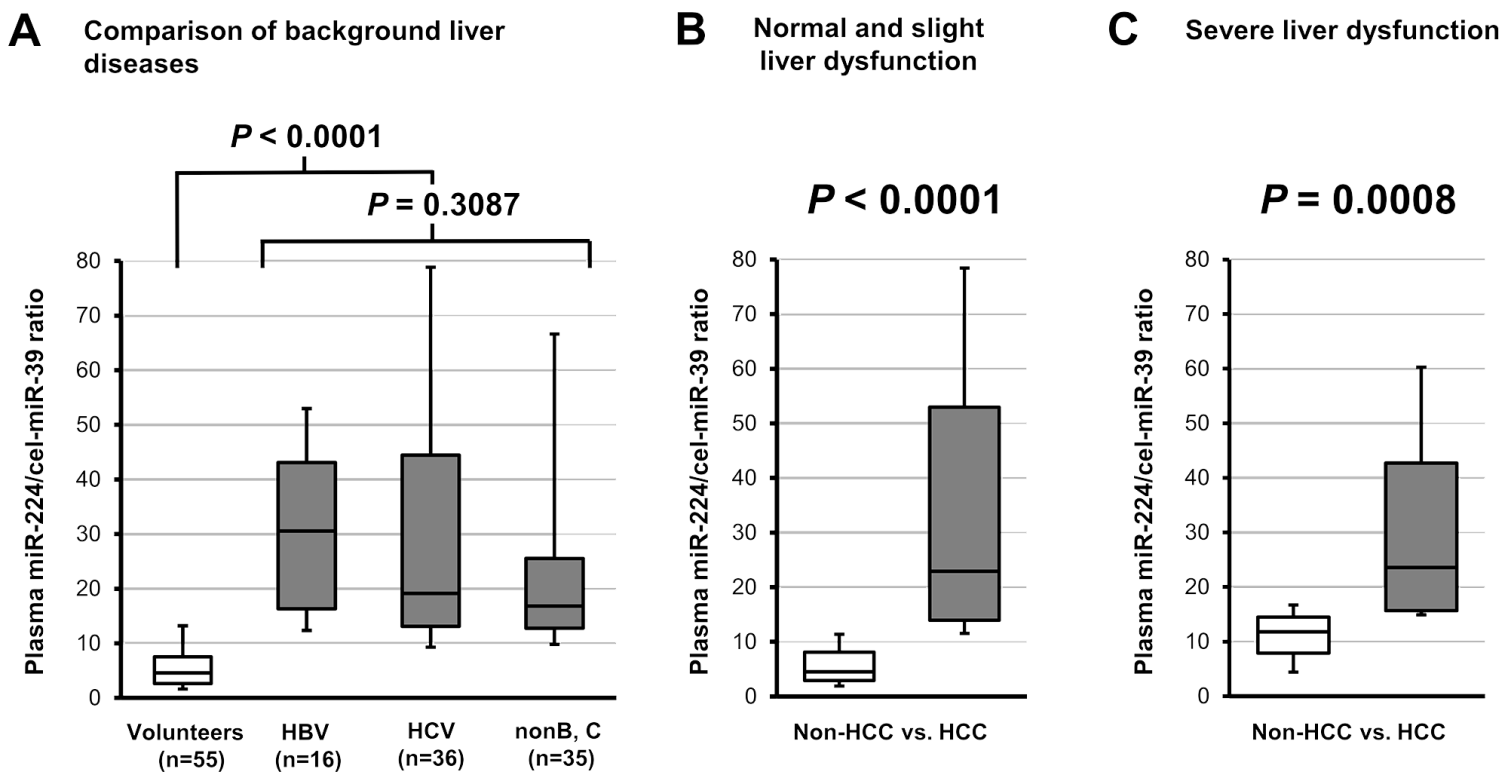
Evaluation of tumor detection based on the plasma miR-224 level, independent of chronic hepatic disease and hepatic function **A.** There were no significant differences in the plasma miR-224 levels among patients with HBV, HCV and other disease in the large-scale analysis (P = 0.3087). **B, C.** The plasma miR-224 levels were significantly higher in the plasma of HCC patients compared with non-HCC outpatients among patients with mild (P < 0.0001) or severe liver dysfunction (P = 0.0008).

**Figure 6:**
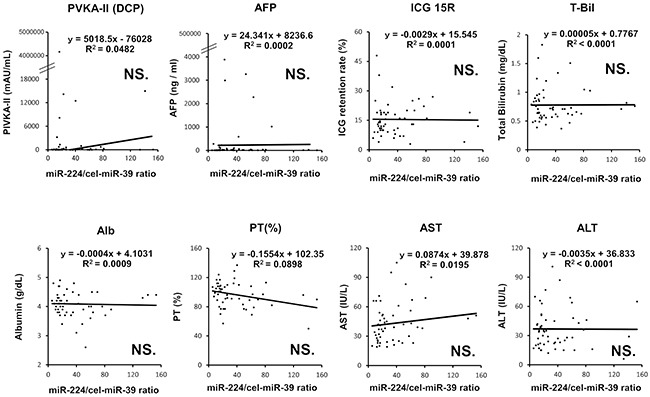
Correlation between the plasma level of miR-224 and conventional serum tumor markers and indicators of hepatic functions in HCC patients No significant correlation was observed between the plasma miR-224 levels and other clinical indicators, such as the tumor makers PIVKA-II (DCP) and AFP, the ICG retention rate (ICG 15R), total bilirubin (T-Bil), albumin (Alb), prothrombin time (PT (%)), aspartate transaminase (AST) and alanine transaminase (ALT).

**Figure 7:**
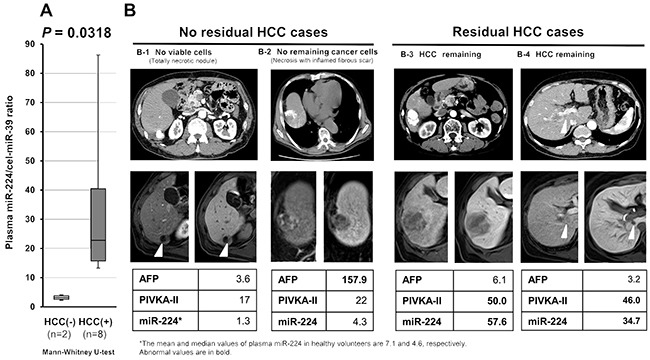
Post-treatment plasma miR-224 level as a sensitive indicator of residual tumor during HCC treatment Current tumor markers and imaging modalities, such as Lipiodol^®^-CT and dynamic MRI, have limitations in the detection of residual tumor after local therapy for HCC. **(A)** The plasma miR-224 level was significantly higher in patients with residual tumors than in patients with no remaining cancer cells (P = 0.0318) **(B)** In these patients, conventional tumor markers and dynamic MRI could not discriminate residual HCC.

The present study was designed according to the following scheme: (1) Selection of appropriate miRNA candidates based on a systematic review of the NCBI database (Figure 1B and Supplementary Table S1); (2) Small-scale analysis of the plasma samples using qRT-PCR to validate the utility of the selected miRNA candidates (Figure 2 and Supplementary Figure S1); (3) Confirmation of higher miR-224 expression in primary HCC tissues and HCC cell lines (Figure 3A and 3B); (4) Large-scale analysis to validate the plasma level of miR-224 (Figure 3C) and two independent-cohorts analyses (Supplementary Figure S2); (5) Evaluation of the clinicopathological features related to plasma miR-224 level (Table 1 and Supplementary Table S2); (6) Evaluation of whether the plasma miR-224 expression levels reflect tumor dynamics (Figure 3D and 3E); (7) Evaluation of whether the plasma miR-224 expression in peripheral blood could be a diagnostic biomarker independent of hepatic function (Figure 5A, 5B, 5C, Figure 6 and Supplementary Figure S3B); and (8) Evaluation of whether the plasma miR-224 expression level could be an indicator of clinical outcome (Figure 7A and 7B); revealing that (9) plasma miR-224 is a novel candidate for HCC screening, monitoring, and evaluating treatment outcome.

**Table 1:** Association between plasma miR-224 level and clinicopathological characteristics in patients with HCC

Variables		n	Plasma miR-224	
			Median	*P* value^a^
Total		87	19.69	
Gender	male	59	17.02	0.1396
	female	28	23.60	
Age (years old)	< 70	32	17.23	0.3963
	70 ≤	55	19.69	
BMI^b^	< 25	60	20.27	0.1364
	25 ≤	27	14.99	
DM^c^	Positive	25	17.22	0.2225
	Negative	62	19.69	
Viral infection (HBV or HCV)	Positive	52	21.90	0.1207
	Negative	35	16.82	
Liver cirrhosis	Positive	28	20.46	0.3848
	Negative	59	17.32	
Pathological Type	Well^d^	23	16.82	0.3519
	Moderately or poorly^e^	64	19.85	
Vascular invasion	Positive	14	16.90	0.4499
	Negative	73	19.69	
Tumor size (mm)	<20	35	14.99	**0.0005**
	20 ≤	52	24.02	
pStage (LCSGJ^f^)	I	30	15.36	**0.0382**
	II-IV	57	20.34	
pStage (TNM: AJCC/UICC^g^)	I	62	19.85	0.4464
	II-IV	25	17.16	
Recurrence	Positive	29	25.22	**0.0027**
	Negative	58	16.21	

a Mann-Whitney U-test, NOTE : Significant values are indicated in bold;

b Body mass index;

c diabetes mellitus;

d well-differentiated hepatocellular carcinoma;

e moderately or poorly differentiated hepatocellular carcinoma;

f Liver Cancer Study Group of Japan;

g American Joint Committee on Cancer/International Union Against Cancer

### Selection of candidate miRNAs from the NCBI database

Using a systematic review of the NCBI database, we selected candidate miRNAs. Figure 1B shows a detailed description of the selection process. A total of 281 relevant full articles related to HCC were retrieved from PubMed up to February 2013. From these reports, we selected 124 candidate miRNAs as shown in Supplementary Table S1. The detailed selection criteria for the candidate miRNAs are described in the Materials and Methods. To identify more sensitive and reliable biomarkers, we focused on miRNAs whose expression levels had been previously validated in HCC tissues. Consequently, 34 miRNAs were excluded. Next, 11 miRNAs previously reported as biomarkers in body fluids were excluded. We limited the target miRNAs to oncogenic miRNAs, as previous studies have revealed that high plasma levels of oncogenic miRNAs might be derived from tumor necrosis, apoptosis and the active release of secretory vesicles, such as exosomes, from cancer cells; thus, these miRNAs in plasma could reflect tumor dynamics in cancer tissues [34–35, 40–41]. Tumor-suppressive miRNAs and functionally unknown miRNAs were excluded in the present study because the origin of these miRNAs is not well known [38]. Consequently, 48 miRNAs were excluded. Furthermore, 27 miRNAs, insufficient for clinical application and reported in only one article, were excluded. After a series of exclusion criteria were applied, we systematically selected four candidate miRNAs: miR-151 [48–49], miR-155 [50–51], miR-191 [52–53], and miR-224 [54–58].

### Small-scale analysis of the plasma levels of four candidate miRNAs in HCC patients and healthy volunteers

We next investigated the plasma levels of the four miRNAs in 20 consecutive HCC patients and 20 healthy volunteers using qRT-PCR as a small-scale analysis. Regarding this cohort, the proportion of background hepatic disease was categorized as 40% of HCC patients with non-B, non-C and 60% of HCC patients with viral etiology. The plasma levels of all candidate miRNAs were significantly higher in HCC patients than in healthy volunteers, the plasma miR-224 level was validated to exhibit the most significant difference (Figure 2). Moreover, the plasma levels of miRNA-151 and miRNA-191 were significantly higher in the plasma of HCC patients with hepatitis B or C than in non-B, non-C patients. Therefore, we excluded these two miRNAs from the candidate miRNAs to select candidates independent of background liver disease with different etiologies (Supplementary Figure S1). Furthermore, we utilized the area under the ROC curve using the Youden index and calculated the value for the AUC for miR-155 and miR-224 [59]. The AUC value for the plasma miR-155 and miR-224 were 0.589 and 0.905, respectively. Thus, we selected the most promising candidate, miR-224, for further analyses.

### Confirmation of higher miR-224 expression in HCC tissues and cell lines

Next, we confirmed the expression level of miR-224 in primary HCC tissues and HCC cell lines compared with normal tissues and the fibroblast cell line WI-38. We used qRT-PCR to determine the expression of miR-224 in paired 12 normal hepatic tissues (Figure 3A) and in the human HCC cell lines, such as JHH, HLE, Hep-G2 and Alexander cells (Figure 3B). The miR-224 expression level was significantly higher in HCC tissues than in normal hepatic tissues (P = 0.0011) (Figure 3A). The expression of miR-224 was significantly higher in the HCC cell lines than in normal hepatic tissues and the fibroblast cell line WI-38 (P = 0.0150) (Figure 3B). These findings strongly suggested that miR-224 expression was highly expressed in HCC, as shown in previous reports [54–58].

### Large-scale analysis of the miR-224 plasma levels in HCC patients

We next validated these observations in large-scale settings. Prior to the analysis of a large number of samples using qRT-PCR, the linearity of qRT-PCR was confirmed using various concentrations of 1 fmol to 0.0001 fmol of each synthetic miRNA, namely, miR-224 (R^2^ = 0.9981) and cel-miR-39 (R^2^ = 0.9977), between the logarithm of the amount of input miRNA and Ct values (Supplementary Figure S3A). Recent reports have demonstrated that some circulating miRNAs might be derived from peripheral blood cells [60]. In the present study, there was no significant correlation between the plasma miR-224 level and the number of any type of peripheral blood cell (Supplementary Figure S3B).

Plasma miR-224 was detected in all samples from the 87 HCC patients and 55 healthy volunteers. Using large validation cohorts, we showed that the plasma level of miR-224 was significantly higher in the HCC patients than in the healthy volunteers (P < 0.0001) (Figure 3C). Furthermore, to detect any cut-off points differentiating cancer patients from healthy volunteers, we utilized the area under the ROC curve using the Youden index [59] and calculated the value for the AUC of the validation cohort. The AUC value for the plasma miR-224 analysis was 0.908. The optimal cut-off point was 8.0 in relative expression using the miR-224/cel-miR-39 ratio with a sensitivity of 93.1%, a specificity of 80.0%, and an accuracy of 88.0%.

Because the validation cohorts comprised samples from two independent institutes, we also examined variations using two independent cohorts. The results were nearly identical in each independent cohort (Supplementary Figure S2). In the two independent cohorts, the miR-224 plasma level was significantly higher in the HCC patients than in the healthy volunteers (P < 0.0001). Additionally, the AUC values for the plasma miR-224 analysis were 0.914 and 0.906, respectively. In the first cohort, the cut-off point was 8.0 in relative expression using the miR-224/cel-miR-39 ratio with a sensitivity of 87.7%, a specificity of 86.3%, and an accuracy of 88.5%. In second cohort, the cut-off point was 8.0 in relative expression using the miR-224/cel-miR-39 ratio with a sensitivity of 87.7%, a specificity of 86.3%, and an accuracy of 87.2%. Similar to the small-scale analysis, the consistency of the results using large-scale validation cohorts and two independent cohorts provided strong evidence that the plasma miR-224 level could satisfactorily distinguish HCC patients from healthy volunteers compared with conventional tumor markers.

Furthermore, to demonstrate the clinical utility, we next compared the plasma miR-224 levels between early stage HCC patients and the healthy volunteers in the validation cohort. We defined stage I HCC as early stage HCC, according to the two staging systems such as the LCSGJ and TNM (AJCC/UICC). The results were almost same in both staging systems, namely, the miR-224 plasma level was significantly higher in stage I HCC patients than in the healthy volunteers (P < 0.0001). The AUC values in the LCSGJ and TNM (AJCC/UICC) staging systems for the plasma miR-224 analysis were 0.888 and 0.899, respectively. These results clearly demonstrated that the plasma miR-224 level could satisfactory distinguish early stage HCC patients form healthy volunteers (Figure 4).

### Evaluation of whether plasma miR-224 expression reflects tumor dynamics

To validate whether the plasma miR-224 level reflects tumor dynamics during the treatment of HCC patients, we first confirmed that the plasma miR-224 levels were significantly correlated with the tumor tissue levels of miR-224 using paired samples (P = 0.0002) (Figure 3D). Second, we evaluated the plasma level of miR-224 in paired samples collected before and almost 1 month after surgery from 8 HCC patients who underwent curative hepatectomy and observed that miR-224 was significantly reduced in these postoperative plasma samples (P = 0.0058) (Figure 3E). These results indicated that the plasma miR-224 level precisely reflects the HCC tissue status and could be used to trace tumor dynamics in HCC patients.

### Evaluation of tumor detection based on plasma miR-224 levels independent of chronic hepatic disease and hepatic function

To demonstrate the clinical utility, we validated the plasma level of miR-224 in HCC patients without associated background liver disease and liver function. As shown in the test-scale analysis (Supplementary Figure S1), there were no significant differences in the plasma miR-224 levels among patients with HBV, HCV and other disease in the large-scale analysis (P = 0.3087) (Figure 5A). The plasma miR-224 levels were significantly higher in HCC patients compared with non-HCC outpatients among individuals with mild (Figure 5B) or severe liver dysfunctions (P = 0.0008) (Figure 5C). Furthermore, no significant correlation was observed between the plasma miR-224 levels and other clinical indicators, such as the tumor makers PIVKA-II (DCP) and AFP, the ICG retention rate (ICG 15R), total bilirubin (T-Bil), albumin(Alb), prothrombin time (PT (%)), aspartate transaminase (AST) and alanine transaminase (ALT) (Figure 6).

### Correlation between the miR-224 plasma levels and the clinicopathological factors in HCC patients

We analyzed whether plasma miR-224 levels were correlated with clinicopathological factors in the validation cohort comprising all 87 consecutive patients with HCC. The results showed that large tumor sizes of more than 20 mm, an advanced pStage (LCSGJ) of more than II and the presence of recurrences were significantly correlated with high levels of plasma miR-224 (P = 0.0005, 0.0382 and 0.0027, respectively), whereas clinical factors, such as gender, age, BMI, diabetes, viral infection and liver cirrhosis, types of recurrence were not correlated with plasma miR-224 levels (Table 1, Supplementary Table S3A). These tendencies were same in two independent cohorts (Supplementary Table S2, S3B).

### Comparison of the minimum tumor detectability between plasma miR-224 and conventional serum tumor markers

Next, we compared the minimum tumor detectability between plasma miR-224 and conventional serum tumor markers, such as PIVKA-II and AFP, in 54 consecutive HCC patients who underwent hepatectomy at Kyoto Prefectural University of Medicine (First Cohort) and 33 consecutive HCC patients who underwent hepatectomy at Kyoto Second Red Cross Hospital (Second Cohort). Regarding the cut-off value of plasma miR-224, the most sensitive cut-off value, determined using the ROC curve to detect the smallest tumor, was used (Supplementary Table S4). As shown in Table 2, the plasma miR-224 level was more sensitive to the presence of smaller tumors than were conventional tumor markers, such as PIVKA-II and AFP. Specifically, the plasma miR-224 level could detect a small tumor of less than 18 mm with highest AUC value. These results were clearly demonstrated in two independent cohorts.

**Table 2:** Comparison of tumor detectability between plasma miR-224 level and conventional serum tumor markers

**First cohort**				
**Biomarkers**	**AUC**	**Minimum detectable tumor size**	**Sensitivity (Tumor <18 mm)**	**Sensitivity (all patients)**
**Plasma miR-224**	0.802	18 mm	80.0%	90.7%
cut-off value: 11*				
**Serum PIVKA-II**	0.741	21 mm	45.0%	62.2%
cut-off value: 40**				
**Serum AFP**	0.475	78 mm	50.0%	40.7%
cut-off value: 20***				

* miR-224/cel-miR-39 ratio,

** mAU/ml,

*** ng/ml

**Table:** 

**Second cohort**				
**Biomarkers**	**AUC**	**Minimum detectable tumor size**	**Sensitivity (Tumor <18 mm)**	**Sensitivity (all patients)**
**Plasma miR-224**	0.731	18mm	63.6%	78.7%
cut-off value: 11*				
**Serum PIVKA-II**	0.595	28mm	50.0%	50.0%
cut-off value: 40**				
**Serum AFP**	0.726	26mm	20.0%	30.3%
cut-off value: 20***				

* miR-224/cel-miR-39 ratio,

** mAU/ml,

*** ng/ml

### Post-treatment plasma miR-224 level as a sensitive indicator of residual tumor

Because current tumor markers and imaging modalities, such as Lipiodol^®^-CT and dynamic MRI, have limitations in the detection of the residual tumor after local therapy for HCC (Figure 7B), we investigated whether plasma miR-224 could sensitively detect residual tumors. We analyzed 10 patients with subsequently resected tumors after local therapies, such as percutaneous ablation therapy and TACE. Pathologically, 2 patients had no remaining cancer cells, whereas the remaining 8 patients had residual tumors. The plasma miR-224 levels were significantly higher in the 8 patients with residual tumors than in patients without remaining cancer cells (P = 0.0318) (Figure 7A). In these patients, conventional tumor markers and dynamic MRI could not discriminate the residual HCC (Figure 7B).

## DISCUSSION

No ideal and generally accepted blood-based molecular biomarkers, the so-called liquid biopsy, for the sensitive cancer screening and monitoring of HCC independent of background hepatic diseases and hepatic function have been identified. In the present study, we selected candidate miRNAs through a systematic review of HCC in the NCBI database and various validation analyses and identified plasma miR-224 as a novel biomarker for HCC diagnosis and treatment. Specifically, the expression level of plasma miR-224 was significantly higher in HCC patients than in healthy volunteers in two independent-cohorts and large-scale analyses (AUC: 0.908). Moreover, the detectability of HCC using plasma miR-224 was excellent and independent of background liver etiologies and the degree of liver dysfunction, even in liver cirrhosis. These findings might facilitate a more sensitive diagnosis and better decision-making during HCC treatment, strongly highlighting the usefulness of miR-224 as a novel and non-invasive liquid-based biomarker for HCC.

Regarding the molecular function of miR-224, the expression of miR-224 is primarily regulated through signaling pathways, such as the NF-κB inflammatory signaling pathway and TGF-β signaling pathways [56, 61]. This molecule is also reported as a master regulator of cell cycle progression and its overexpression results in G1/S checkpoint release followed by accelerated cell growth [62]. Recent studies have shown that miR-224 is more highly expressed in not only HCC but also esophageal cancer, non-small cell lung cancer, colorectal cancer, cervical cancer and glioma, and this molecule has a proliferative effect on these cancer cells through direct interactions with various tumor-suppressor genes, such as CAMKK2, ADAM17, Homeobox D 10 and RKIP [63–68]. Moreover, the clinical relevance of miR-224 had been reported as an indicator of chemoresistance to cisplatin, methotrexate and R-CHOP, radiation sensitivity and a prognostic factor of sorafenib-treated patients [69–74]. These findings strongly suggest that miR-224 might have a pivotal role in carcinogenesis and the development of HCC tumors. Thus, we hypothesized that the upregulation of plasma miR-224 might be associated with the aggressive clinical features and poor outcomes of HCC. Indeed, a high plasma miR-224 level was significantly associated with larger tumor size (P = 0.0005), higher pathological stage (P = 0.0382) and recurrence rate (P = 0.0027). Previous report already demonstrated the association between tumor size and recurrence, therefore, our results suggested that a high plasma miR-224 level is associated with tumor development [75]. Thus, plasma miR-224 levels might be a useful molecular biomarker for primary and recurrent HCC detection.

The most striking finding of the present study was that plasma miR-224 levels could discriminate HCC from non-HCC patients under various hepatic diseases, such as viral hepatitis, alcoholic liver disease, fatty liver, NAFLD, AIH, liver cirrhosis, NASH and primary PBC (Figure 5B and 5C). Indeed, there was no significant correlation between the plasma miR-224 levels and various indicators of hepatic function, such as platelet, albumin, T-Bil, Alb, PT (%), AST, ALT and ICG 15R (Figure 6 and Supplementary Figure S3B). In clinical settings, conventional tumor markers lack sufficient sensitivity and specificity to facilitate HCC detection, and previous reported biomarkers for HCC often reflect background liver disease and hepatic function [76–80]. Whereas, the effectiveness of plasma miR-224 was demonstrated to facilitate the sensitive HCC detection of smaller tumors of less than 18 mm (AUC: 0.802). The sensitivity for HCC detection in plasma miR-224 was 78.7-90.7% (Table 2). This result indicates that the sensitivity of plasma miR-224 was not inferior to that of recent imaging modalities such as ultrasonography, computed tomography (CT), gadolinium ethoxybenzyl diethlenetriamine pentaacetic acid-enhanced liver magnetic resonance imaging (Gd-EOB-DTPA-enhanced MRI), their sensitivities were 60.5%, 76.0% and 88.5%, respectively [81–83]. Moreover, plasma miR-224 may be an indicator of residual tumor in non-surgical treatment such as percutaneous ablation therapy and/or TACE although this was preliminary result because the number of cases was small (Figure 7). These findings strongly suggest that plasma miR-224 can inform HCC treatment as a next-generation molecular blood-based biomarker.

Regarding plasma miRNAs, previous studies concerning blood miRNA profiles revealed that the majority of circulating miRNAs were co-fractionated with plasma protein complexes [26]. Moreover, compared to serum, plasma might retain higher protein levels, including those of coagulant-related proteins. These finding prompt us to consistently use plasma miRNAs in future clinical application [25, 34–44]. Indeed, miRNA profiles and miRNA concentrations were considerably different between serum and plasma. Namely, this phenomenon was detected in studies of the diagnostic blood-based miRNA candidates in ESCC [41, 84] and pancreatic cancer [44, 85]. These findings strongly suggest that we should fully consider the kinds of blood samples such as serum, plasma and all blood for better clinical application of miRNAs as a blood-based biomarker. Although clinical utility of serum miR-224 in HCC was reported [86–88], our study might be also valuable as a first report to demonstrate the diagnostic utility of plasma miR-224 in HCC, independent of hepatic status.

This study is the first to demonstrate the diagnostic utility of plasma miR-224 in HCC, independent of hepatic status. However, many issues must be addressed before these findings can be translated into a clinically useful and non-invasive screening strategy for HCC patients. Namely, more sensitive candidate miRNAs should be identified as liquid-based biomarkers for the diagnosis and monitoring of HCC using strategies with different body fluids and high-throughput platforms, such as next-generation sequencing or digital PCR-based approaches. Furthermore, we will prospectively confirm the usefulness of plasma miR-224 in a larger number of patients with cancers, if possible, including not only HCC but also other types of cancer with miR-224 overexpression such as esophageal cancer, non-small cell lung cancer, colorectal cancer, cervical cancer and glioma. These strategies are currently under evaluation.

## MATERIALS AND METHODS

### Patients and samples

This study was approved by the Institutional Review Board of both Kyoto Prefectural University of Medicine and Kyoto Second Red Cross Hospital, Kyoto, Japan, and each subject provided signed informed consent. Between January 2010 and December 2014, a total of 211 plasma samples were collected, including 109 samples from HCC patients, 75 samples from healthy volunteers and 27 samples from outpatients with chronic liver disease. The 109 plasma samples from HCC patients were obtained from Kyoto Prefectural University of Medicine and comprised 20 small-scale samples, 54 validation samples and 2 samples from patients who underwent resection following pretreatment, such as percutaneous ablation therapy and/or trans-catheter arterial chemoembolization therapy (TACE), and in whom no remaining cancer cells were found (1st cohort), and the 33 validation samples were obtained from Kyoto Second Red Cross Hospital (2nd cohort). The 27 plasma samples from outpatients with chronic liver disease were obtained from Kyoto Prefectural University of Medicine. These samples were divided into two groups: 16 outpatients diagnosed with viral hepatitis, alcoholic liver disease, fatty liver, non-alcoholic fatty liver disease (NAFLD) and autoimmune hepatitis (AIH), defined as “mild liver dysfunction outpatients,” and 11 outpatients diagnosed with liver cirrhosis or NASH (non-alcoholic steatohepatitis) or primary biliary cirrhosis (PBC) were defined as “severe liver dysfunction outpatients.” The 75 samples from healthy volunteers comprised 20 small-scale samples and 33 validation samples from Kyoto Prefectural University of Medicine (1st cohort) and 22 validation samples from Kyoto Second Red Cross Hospital (2nd cohort). These healthy volunteers included medical personnel and patients with benign diseases, such as cholelithiasis and inguinal hernia. The patients with benign diseases and outpatients with mild or severe liver dysfunction underwent medical examinations, including computed tomography and endoscopy, and were confirmed as not having HCC or any cancerous diseases.

Concerning the tissue samples from the patients who underwent hepatic resection, a total of 24 HCC specimens and 17 normal hepatic specimens were collected. Half (12) of the HCC specimens were paired with validation plasma samples to determine a correlation, whereas the remaining (12) HCC specimens were paired with adjacent normal hepatic specimens resected from the same patients. A total of 5 adjacent normal hepatic specimens were obtained from patients who underwent hepatic resection for HCC and compared with HCC cell lines.

Peripheral blood (7 ml) was obtained from each patient at the time of diagnosis or before surgery and from the healthy volunteers. The blood was transferred into sodium heparin tubes (BD Vacutainer, Franklin Lakes, New Jersey, the United States) and immediately subjected to a three-spin protocol (1500 r.p.m. for 30 min, 3000 r.p.m. for 5 min, and 4500 r.p.m. for 5 min) to prevent contamination with cellular nucleic acids. The plasma was collected and stored at -80°C until further processing. The resected specimens were fixed in formalin and embedded in paraffin for pathological diagnosis. Histological evaluation was performed for tissues adjacent to specimens according to the criteria of the World Health Organization. In all cases, at least two pathologists agreed with pathological observations and confirmed the diagnosis. The tumor stages were assessed according to General Rules for the Clinical and Pathological Study of Primary Liver Cancer by Liver Cancer Study Group of Japan (LCSGJ) [89] and TNM classification system by American Joint Committee on Cancer (AJCC) and International Union Against Cancer (UICC) [90].

### RNA extraction

Total RNA was extracted from 400 μl of plasma using a mirVana PARIS Kit (Ambion, Austin, TX) and eluted into 100 μl of preheated (95°C) Elution Solution according to the manufacturer's protocol. A volume of 400 μl of plasma was used as the common denominator in each microarray analysis; thus, there was no definite internal control in the plasma miRNA analyses as shown in previous studies [34–41]. Total RNA was also extracted from four 15-μm-thick slices of the formalin-fixed and paraffin-embedded tissue samples (60 μm thickness) using a Recover All Total Nucleic Acid Isolation Kit (Ambion) and subsequently eluted into 60 μl of Elution Solution according to the manufacturer's instructions.

### A systematic review of the NCBI database to select candidate miRNAs

We performed a systematic review of the NCBI database to identify novel plasma biomarkers of miRNA in patients with HCC (Figure 1B and Supplementary Table S1). We searched for all studies related to HCC miRNAs in PubMed up to February 2013. This search was based on the key terms ‘HCC’ and ‘microRNA.’ Any meeting abstracts not accompanied by full articles and other incomplete and non-English articles were excluded. The candidate miRNAs had to meet the following criteria: I) Candidate miRNAs whose overexpression was validated in HCC tissues compared with normal liver or peritumoral non-cancerous tissues; II) Candidate miRNAs not previously reported as biomarkers in body fluids, such as plasma, serum, urine and semen; III) Candidate miRNAs demonstrated to have oncogenic roles (functionally unknown or tumor suppressive miRNAs were excluded); and IV) Candidate miRNAs reported several times with sufficient data in HCC. Two authors independently reviewed all articles identified in this search using these criteria, and a third author resolved any discrepancies.

### Quantification of miRNA by qRT-PCR

The amounts of miRNAs were quantified by qRT-PCR using a human TaqMan MicroRNA Assay Kit (Applied Biosystems, Foster City, CA). A reverse transcription reaction was performed using a TaqMan MicroRNA Reverse Transcription Kit (Applied Biosystems) in 5 μl of solution containing 1.67 μl of extracted RNA, 0.05 μl of 100 mM dNTPs, 0.33 μl of Multiscribe Reverse Transcriptase (50 U μl^-1^), 0.5 μl of 10× Reverse Transcription Buffer, 0.06 μl of RNase inhibitor (20 U μl^-1^), 1 μl of gene-specific primer (hsa- miR-151, Assay ID: 000596; hsa-miR-155, Assay ID: 000479; hsa-miR-191, Assay ID: 000490; hsa-miR-224, Assay ID: 000599; cel-miR-39, Assay ID: 000200; and RNU6B, Assay ID: 001093), and 1.39 μl of nuclease-free water. To synthesize cDNA, the reaction mixtures were incubated at 16°C for 30 min, 42°C for 30 min, and 85°C for 5 min, after which the reactions were held at 4°C. Next, 0.67 μl of cDNA was amplified using 5 μl of TaqMan 2× Universal PCR Master Mix with no AmpErase UNG (Applied Biosystems), 0.5 μl of gene-specific primers/probe, and 3.83 μl of nuclease-free water in a final volume of 10 μl. Quantitative PCR was run on a StepOnePlus PCR system (Applied Biosystems), and the reaction mixtures were incubated at 95°C for 10 min, followed by 40 cycles of 95°C for 15 sec and 60°C for 1 min. The cycle threshold (Ct) values were calculated using StepOne Software v2.0 (Applied Biosystems).

As previously reported [22], we used an approach to data normalization based on spiking the sample with a synthetic RNA oligonucleotide, cel-miR-39, which does not exist in the human genome. *C. elegans* cel-miR-39 was purchased as a custom-made RNA oligonucleotide (Qiagen, Valencia, CA). The oligo used for spiking, as a mixture of 25 fmol of the oligonucleotide in a total volume of 5 μl of water, was introduced after the addition of 2X Denaturing Solution (Ambion) to the plasma or serum sample to avoid degradation by endogenous plasma RNases. As a control for each RNA sample, cel-miR-39 was used for TaqMan qRT-PCR assays (Applied Biosystems) as previously described. The data were normalized across samples using the 2^-ΔΔCt^ method relative to cel-miR-39. The miRNA expression from tissue samples and cultured cells was normalized using the 2^-ΔΔCt^ method relative to U6 small nuclear RNA (RNU6B). The ΔCt was calculated after subtracting the Ct values of cel-miR-39 or RNU6B from those of the miRNAs of interest. The ΔΔCt was subsequently calculated after subtracting the mean of ΔCt of the plasma of healthy volunteers or normal pancreatic tissue samples from the ΔCt of HCC tissues. Changes in gene expression were calculated using the equation 2^-ΔΔCt^ [91–92].

### Culture of HCC cell lines

The HCC cell lines JHH-6, HLE and Alexander were purchased from the JCRB Cell Bank (Osaka, Japan), and HepG2 was purchased from the RIKEN cell bank (Tsukuba, JAPAN) and cultured in Roswell Park Memorial Institute (RPMI)-1640 medium (Sigma, St Louis, MO), Dulbecco's Modified Eagle Medium (DMEM, Nacalai Tesque, JAPAN) and William's Medium E (LANZA, Switzerland) supplemented with 10% FBS (Trace Scientific, Melbourne, Australia). All cell lines were cultured in 5% carbon dioxide at 37°C in a humidified chamber.

### Statistical analysis

The Mann-Whitney U-test for unpaired data from plasma or tissue samples was performed. The Kruskal-Wallis H-test was also used to compare more than two groups. The Wilcoxon test was used to compare the paired HCC and adjacent normal hepatic tissue samples and the paired plasma samples obtained before and at 1 month after hepatectomy. The Chi-square test or Fisher's exact probability test was used to evaluate correlations between the results for the plasma miRNA levels and clinicopathological factors. A P-value < 0.05 was considered statistically significant.

Receiver-operating characteristic curves and the area under the ROC curve were used to assess the feasibility of using plasma miRNA as a diagnostic tool for detecting HCC. The Youden index was used to determine the cut-off value for the plasma miRNA levels [59].

## SUPPLEMENTARY MATERIALS FIGURES AND TABLES




