# Supplementary material for: Circulating microRNA profiles in plasma: identification of miR-224 as a novel diagnostic biomarker in hepatocellular carcinoma independent of hepatic function
Source: Oncotarget. 2016 Jul 22;7(33):53820–36. doi: 10.18632/oncotarget.10781 (PMC5288224; doi:10.18632/oncotarget.10781)
Supplement: Supplementary file 1 [file oncotarget-07-53820-s001.pdf]

Circulating microRNA profiles in plasma: identification of miR-224 as a novel diagnostic biomarker in hepatocellular carcinoma independent of hepatic function

SUPPLEMENTARY FIGURES AND TABLES

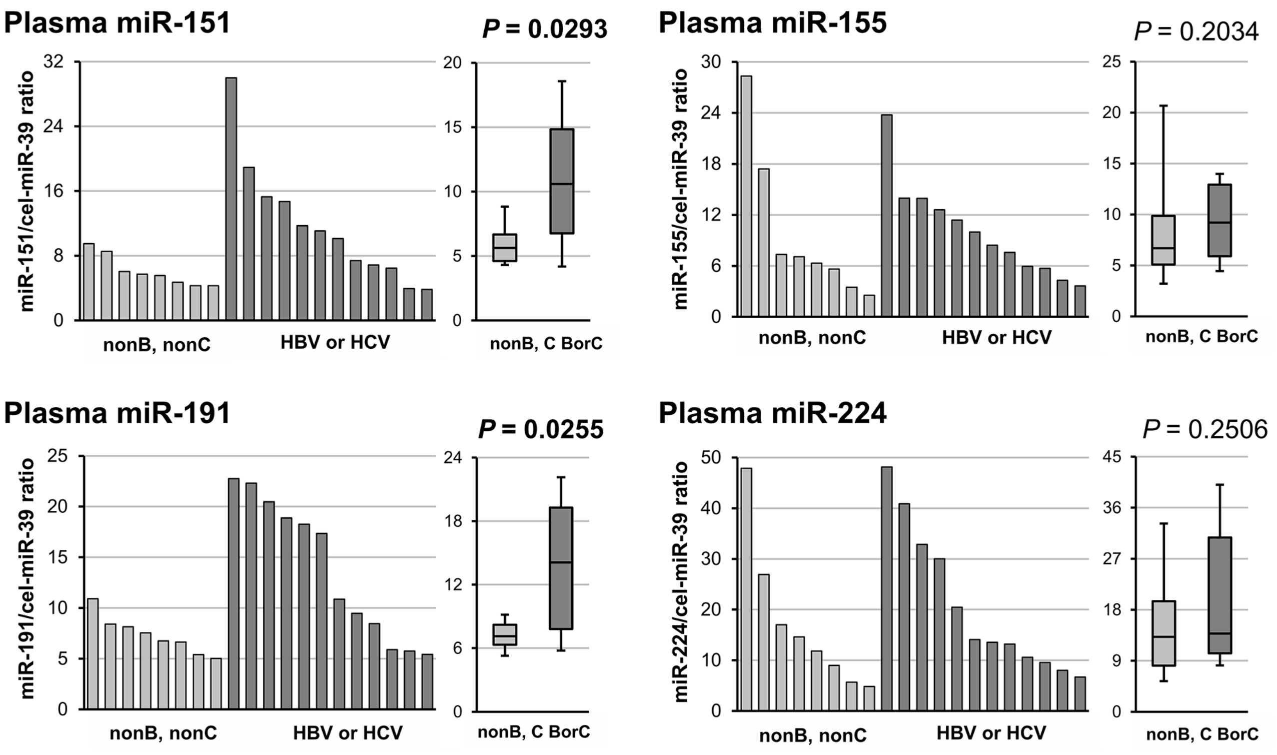

Supplementary Figure S1: Small-scale analyses comparing the plasma levels of four candidate miRNAs between HCC patients with viral etiology and no viral etiology. Plasma levels of the four candidate miRNAs in 20 HCC patients were analyzed using qRT-PCR, from the viewpoint of background hepatic disease. The plasma levels of miRNA-151 and miRNA-191 were significantly higher in the plasma of HCC patients with hepatitis B or C than in non-B, non-C patients.

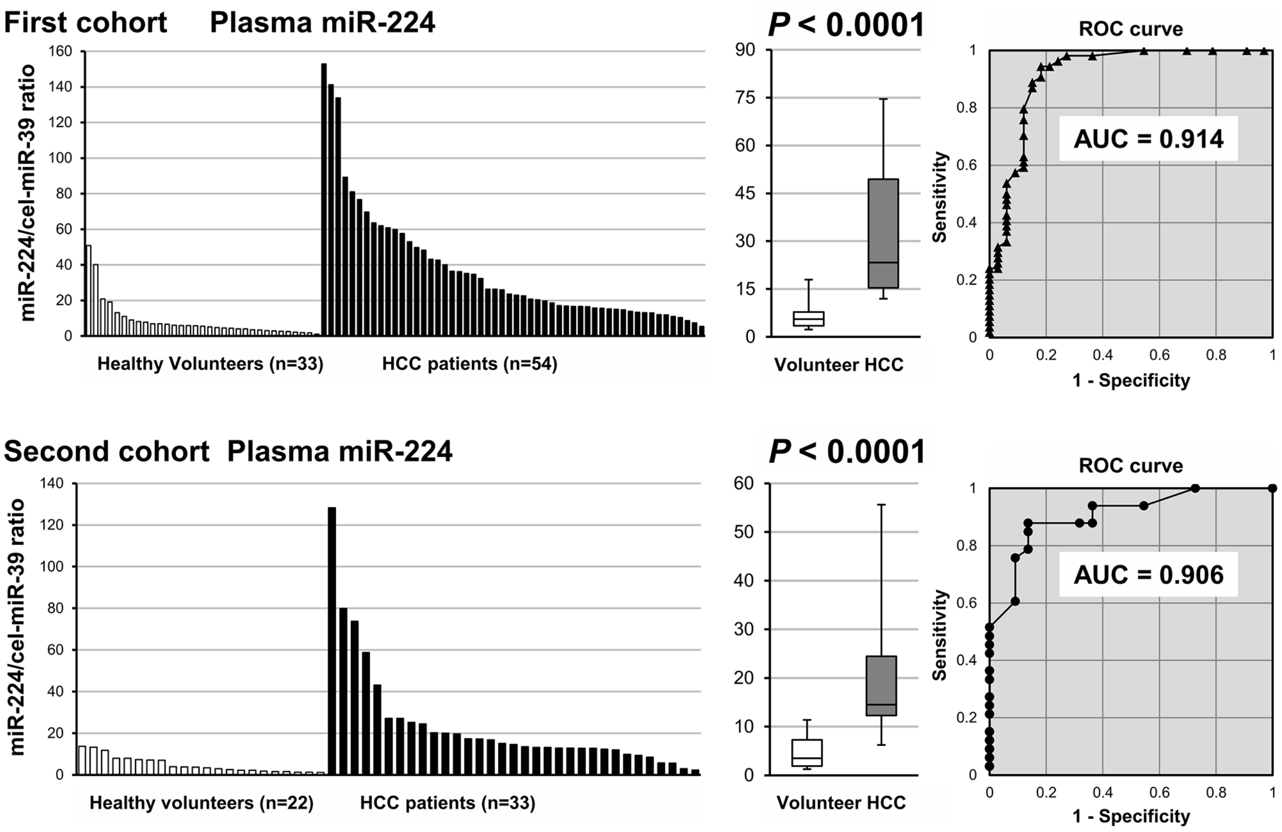

Supplementary Figure S2: The high level of plasma miR-224 was validated in HCC patients in two independent cohorts. The validation cohorts comprised samples from two independent institutes. In the two independent cohorts, the miR-224 plasma level was significantly higher in the HCC patients than in the healthy volunteers ($P < 0.0001$). The results were nearly identical in each independent cohort and similar to large-scale validation cohorts.

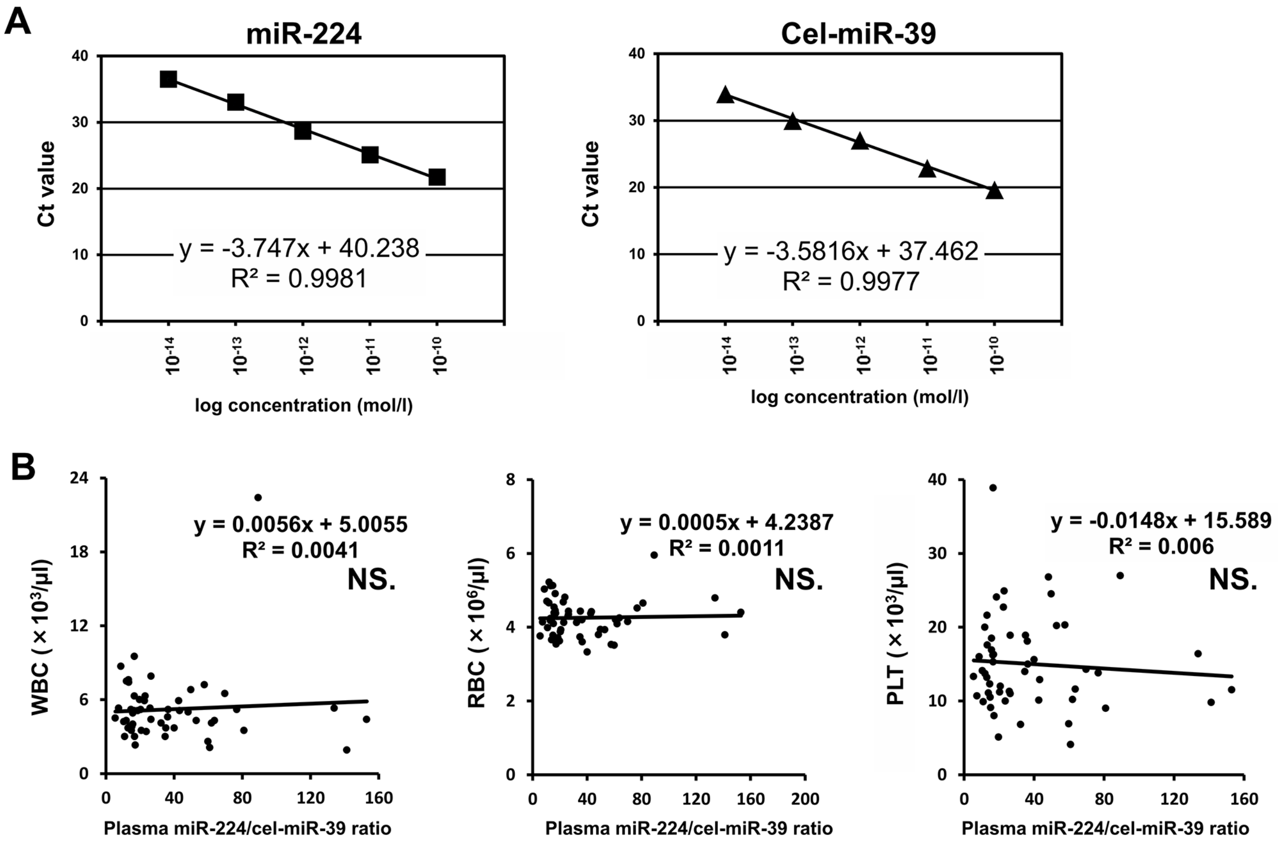

Supplementary Figure S3: A. Standard curves of miR-224 and cel-miR-39 using synthetic miRNAs. Ten-fold serial dilutions of synthetic miRNA were used to generate the standard curves. Linearity was confirmed within these concentrations. **B.** Evaluation of the correlation between the plasma level of miR-224 and peripheral blood cells. There were no significant correlation between the plasma miR-224 level and the number of any type of peripheral blood cell.

Supplementary Table S1: All candidate miRNAs and selected process

See Supplementary File 1

Supplementary Table S2: Association between plasma miR-224 level and clinicopathological characteristics in patients with HCC

Variables		First cohort			Second cohort		
		n	Plasma miR-224		n	Plasma miR-224	
			Median	*P* value[a]		Median	*P* value[a]
Total		54	23.26		33	14.50	
Gender	male	35	20.79	0.1408	24	14.77	0.3825
	female	19	34.75		9	13.13	
Age (years old)	< 70	22	22.74	0.4377	10	12.98	0.2941
	70 ≤	32	29.37		23	15.05	
BMI[b]	< 25	38	26.14	0.2931	22	17.07	0.1565
	25 ≤	16	18.72		11	12.86	
DM[c]	Positive	13	22.55	0.3116	12	14.96	0.4633
	Negative	41	23.60		21	14.50	
Viral infection (HBV or HCV)	Positive	37	26.37	0.4890	15	20.01	0.1609
	Negative	17	22.55		18	14.00	
Liver cirrhosis	Positive	17	22.93	0.4963	11	13.13	0.3886
	Negative	37	23.60		22	14.77	
Pathological Type	Well[d]	12	21.67	0.0699	11	12.68	0.0781
	Moderately or poorly[e]	42	60.37		22	16.13	
Vascular invasion	Positive	9	22.93	0.3529	5	13.50	0.4017
	Negative	45	25.92		28	14.77	
Tumor size (mm)	< 20	23	17.16	**0.0066**	12	12.15	**0.0060**
	20 ≤	31	36.21		21	19.69	
pStage	I	21	18.61	**0.0465**	9	12.26	0.0578
(LCSGJ[f])	II-IV	33	25.92		24	17.00	
pStage	I	38	23.58	0.4702	24	16.13	0.3530
(TNM:AJCC/UICC[g])	II-IV	16	23.27		9	13.50	
Recurrence	Positive	19	34.75	**0.0380**	10	22.22	**0.0232**
	Negative	35	18.61		23	13.10	

a, Mann-Whitney U-test, NOTE : Significant values are indicated in bold; b, Body mass index; c, diabetes mellitus; d, well-differentiated hepatocellular carcinoma; e, moderately or poorly differentiated hepatocellular carcinoma; f, Liver Cancer Study Group of Japan; g, American Joint Committee on Cancer / International Union Against Cancer

Supplementary Table S3A: Association between plasma miR-224 level and types of recurrence in patients with HCC

Recurrence pattern (Validation cohort)	n	Plasma miR-224	
		Median	*P* value[a]
Total	29	25.22	
Intrahepatic recurrence (≤3 nodules)	20	25.79	0.1516
Multiple intrahepatic recurrence (>3 nodules)	7	40.05	
Extrahepatic recurrence	2	16.03	

a, The Kruskal-Wallis H-test

Supplementary Table S3B: Two independent cohort analysis

Recurrence pattern	First cohort			Second cohort		
	n	Plasma miR-224		n	Plasma miR-224	
		Median	*P* value[a]		Median	*P* value[a]
Total	19	34.75		10	22.22	
Intrahepatic recurrence (≤3 nodules)	14	35.47	0.1029	6	22.22	0.3041
Multiple intrahepatic recurrence (>3 nodules)	4	31.30		3	79.90	
Extrahepatic recurrence	1	15.25		1	16.82	

a, The Kruskal-Wallis H-test

Supplementary Table S4: The cut-off values of plasma miR-224 in minimum detectable tumor

Plasma miR-224 cut-off value*	AUC	
	First cohort	Second cohort
9	0.758	0.635
10	0.740	0.686
11	0.802	0.731
12	0.689	0.702
13	0.712	0.672

*miR-224/cel-miR-39 ratio
