# Supplementary material for: Circulating microRNA profiles in plasma: identification of miR-224 as a novel diagnostic biomarker in hepatocellular carcinoma independent of hepatic function
Source: Oncotarget. 2016 Jul 22;7(33):53820–36. doi: 10.18632/oncotarget.10781 (PMC5288224; doi:10.18632/oncotarget.10781)
Supplement: Supplementary file 2 [file oncotarget-07-53820-s002.docx]

**Supplementary Table S1.　All candidate miRNAs and selected process**

| **candidate miRNA** | **criterion 1**  (validated in tissue) | **criterion 2**  (not reported in body fluid) | **criterion 3**  (oncogenic role) | **criterion 4**  (sufficient data) |
| --- | --- | --- | --- | --- |
| **1** | positive | positive | negative | previously excluded |
| **7** | positive | positive | negative | previously excluded |
| **10a** | positive | positive | negative | previously excluded |
| **10b** | negative | previously excluded | previously excluded | previously excluded |
| **15** | positive | positive | negative | previously excluded |
| **15b** | positive | negative | previously excluded | previously excluded |
| **16** | positive | negative | previously excluded | previously excluded |
| **17** | negative | previously excluded | previously excluded | previously excluded |
| **17-5p** | positive | negative | previously excluded | previously excluded |
| **18** | positive | positive | positive | negative |
| **18a** | negative | previously excluded | previously excluded | previously excluded |
| **18b** | positive | positive | positive | negative |
| **21** | positive | negative | previously excluded | previously excluded |
| **22** | positive | positive | negative | previously excluded |
| **23a** | positive | positive | positive | negative |
| **23b** | negative | previously excluded | previously excluded | previously excluded |
| **25** | negative | previously excluded | previously excluded | previously excluded |
| **26a** | positive | negative | previously excluded | previously excluded |
| **26b** | positive | positive | negative | previously excluded |
| **27a** | negative | previously excluded | previously excluded | previously excluded |
| **29** | positive | positive | negative | previously excluded |
| **29a** | positive | positive | negative | previously excluded |
| **29a-5p** | positive | positive | positive | negative |
| **29b** | positive | positive | negative | previously excluded |
| **29c** | positive | positive | negative | previously excluded |
| **30d** | positive | positive | positive | negative |
| **33** | positive | positive | negative | previously excluded |
| **34a** | positive | positive | negative | previously excluded |
| **92** | positive | positive | positive | negative |
| **92a** | negative | previously excluded | previously excluded | previously excluded |
| **96** | positive | positive | positive | negative |
| **98** | negative | previously excluded | previously excluded | previously excluded |
| **99a** | positive | positive | negative | previously excluded |
| **100** | positive | positive | negative | previously excluded |
| **101** | positive | positive | negative | previously excluded |
| **106b-25** | negative | previously excluded | previously excluded | previously excluded |
| **107** | negative | previously excluded | previously excluded | previously excluded |
| **108** | negative | previously excluded | previously excluded | previously excluded |
| **122** | positive | negative | previously excluded | previously excluded |
| **122a** | positive | positive | negative | previously excluded |
| **124** | positive | positive | negative | previously excluded |
| **124a-2** | negative | previously excluded | previously excluded | previously excluded |
| **125** | positive | positive | negative | previously excluded |
| **125a** | positive | positive | negative | previously excluded |
| **125b** | positive | positive | negative | previously excluded |
| **129-2** | positive | negative | previously excluded | previously excluded |
| **130a** | positive | positive | positive | negative |
| **130b** | negative | previously excluded | previously excluded | previously excluded |
| **132** | positive | positive | negative | previously excluded |
| **135a** | positive | positive | positive | negative |
| **136** | positive | positive | negative | previously excluded |
| **139** | positive | positive | negative | previously excluded |
| **139-5p** | negative | previously excluded | previously excluded | previously excluded |
| **140-5p** | positive | positive | negative | previously excluded |
| **141** | negative | previously excluded | previously excluded | previously excluded |
| **142** | negative | previously excluded | previously excluded | previously excluded |
| **143** | negative | previously excluded | previously excluded | previously excluded |
| **145** | negative | previously excluded | previously excluded | previously excluded |
| **148a** | positive | positive | negative | previously excluded |
| **150** | negative | previously excluded | previously excluded | previously excluded |
| **151** | positive | positive | positive | positive |
| **155** | positive | positive | positive | positive |
| **161** | negative | previously excluded | previously excluded | previously excluded |
| **181a-1** | negative | previously excluded | previously excluded | previously excluded |
| **181a-2** | negative | previously excluded | previously excluded | previously excluded |
| **181b** | negative | previously excluded | previously excluded | previously excluded |
| **181c** | negative | previously excluded | previously excluded | previously excluded |
| **182** | positive | positive | positive | negative |
| **191** | positive | positive | positive | positive |
| **192** | negative | previously excluded | previously excluded | previously excluded |
| **193b** | positive | positive | negative | previously excluded |
| **195** | negative | previously excluded | previously excluded | previously excluded |
| **198** | positive | positive | negative | previously excluded |
| **199** | negative | previously excluded | previously excluded | previously excluded |
| **199a** | positive | positive | negative | previously excluded |
| **199a-3p** | positive | positive | negative | previously excluded |
| **199a-5p** | positive | positive | negative | previously excluded |
| **199b-3p** | positive | positive | negative | previously excluded |
| **200a** | positive | positive | negative | previously excluded |
| **200b** | negative | previously excluded | previously excluded | previously excluded |
| **200c** | negative | previously excluded | previously excluded | previously excluded |
| **203** | positive | positive | negative | previously excluded |
| **210** | positive | positive | positive | negative |
| **214** | positive | positive | negative | previously excluded |
| **216a** | positive | positive | positive | negative |
| **219-5p** | positive | positive | negative | previously excluded |
| **221** | positive | negative | previously excluded | previously excluded |
| **222** | negative | previously excluded | previously excluded | previously excluded |
| **223** | positive | negative | previously excluded | previously excluded |
| **224** | positive | positive | positive | positive |
| **235** | negative | previously excluded | previously excluded | previously excluded |
| **301a** | positive | positive | positive | negative |
| **335** | positive | positive | negative | previously excluded |
| **338-3p** | positive | positive | negative | previously excluded |
| **352** | negative | previously excluded | previously excluded | previously excluded |
| **372** | positive | positive | positive | negative |
| **373** | positive | positive | positive | negative |
| **375** | positive | negative | previously excluded | previously excluded |
| **376a** | positive | positive | negative | previously excluded |
| **423-3p** | positive | positive | positive | negative |
| **450a** | positive | positive | negative | previously excluded |
| **485-3p** | positive | positive | positive | negative |
| **490-3p** | positive | positive | positive | negative |
| **495** | positive | positive | positive | negative |
| **503** | positive | positive | negative | previously excluded |
| **519d** | positive | positive | positive | negative |
| **520b** | positive | positive | negative | previously excluded |
| **520e** | positive | positive | negative | previously excluded |
| **550a** | positive | positive | positive | negative |
| **602** | positive | positive | positive | negative |
| **615-5p** | positive | positive | positive | negative |
| **636** | positive | positive | negative | previously excluded |
| **637** | positive | positive | negative | previously excluded |
| **650** | negative | previously excluded | previously excluded | previously excluded |
| **657** | positive | positive | positive | negative |
| **664** | positive | positive | positive | negative |
| **885-5p** | negative | previously excluded | previously excluded | previously excluded |
| **1271** | positive | positive | negative | previously excluded |
| **1323** | positive | positive | positive | negative |
| **let-7a** | positive | positive | negative | previously excluded |
| **let-7c** | positive | positive | negative | previously excluded |
| **let-7f** | positive | negative | previously excluded | previously excluded |
| **let-7g** | positive | positive | negative | previously excluded |
| **Lin-41** | positive | positive | positive | negative |
